# 3D Image Acquisition System Based on Shape from Focus Technique

**DOI:** 10.3390/s130405040

**Published:** 2013-04-15

**Authors:** Bastien Billiot, Frédéric Cointault, Ludovic Journaux, Jean-Claude Simon, Pierre Gouton

**Affiliations:** 1 Laboratoire Electronique, Informatique et Image, Université de Bourgogne, BP 47870, 21078 Dijon Cedex, France; E-Mails: l.journaux@agrosupdijon.fr (L.J.); jc.simon@agrosupdijon.fr (J.-C.S.); pgouton@u-bourgogne.fr (P.G.); 2 Agrosup Dijon, 26 boulevard Docteur Petitjean, BP 87999, 21079 Dijon Cedex, France; E-Mail: f.cointault@agrosupdijon.fr

**Keywords:** 3D image acquisition system, shape from focus, focus measure, agronomic scenes

## Abstract

This paper describes the design of a 3D image acquisition system dedicated to natural complex scenes composed of randomly distributed objects with spatial discontinuities. In agronomic sciences, the 3D acquisition of natural scene is difficult due to the complex nature of the scenes. Our system is based on the Shape from Focus technique initially used in the microscopic domain. We propose to adapt this technique to the macroscopic domain and we detail the system as well as the image processing used to perform such technique. The Shape from Focus technique is a monocular and passive 3D acquisition method that resolves the occlusion problem affecting the multi-cameras systems. Indeed, this problem occurs frequently in natural complex scenes like agronomic scenes. The depth information is obtained by acting on optical parameters and mainly the depth of field. A focus measure is applied on a 2D image stack previously acquired by the system. When this focus measure is performed, we can create the depth map of the scene.

## 1. Introduction

In order to optimize crop management and take into account the intra-parcel variability (we consider a parcel as heterogeneous), the concept of precision agriculture has been developed over the past thirty years. It consists in a localized crop management using new technologies such as computing, imaging, electronics, *etc.* Based on the data acquired by precision agriculture techniques, Smart Farming is booming because of the increasing amount of such data. Indeed, its goal is the fusion and management of all data, especially from imagery, to optimize the production process. Two types of imagery can be used : proxy-detection and remote sensing. The conception of a proxy-detection system is motivated by the need of better resolution, precision, temporality and lower cost. The advantages of such systems are to provide some information such as the presence of diseases, recognition of the type of plant or yield estimation. The use of computer vision techniques allows us to obtain this information automatically with objective measurement in contrast with the difficulty and subjectivity of visual or manual acquisition. The use of vision systems in two dimensions does not allow us to obtain some particular characteristics. In fact, the parameters that require depth information of the scene, like growth estimation or the determination of leaf volume, are impossible to obtain. The design of a 3D acquisition system is required in order to obtain new parameters related to crops. There are numerous 3D acquisition techniques and they have been the subject of active research for several decades. The overall principle is to determine the shape and structure of a scene from the analysis of the acquired images. The representation of depth information will depend on the type of acquisition technique used, e.g., 3D mesh representation or depth map where a color code corresponds to the position of each pixel in space relative to the acquisition system. This paper begins by presenting the selected 3D acquisition technique and the reasons of such a choice. Then, the prototype is described including the successive measurement and processing of acquired images to provide a depth map. Finally, a conclusion on the contribution of this technique and future work are detailed.

## 2. Background

### 2.1. 3D Acquisition Techniques

Initially, different tests for 3D reconstruction were performed using a Konica Minolta scanner. This type of device uses the principle of laser triangulation to get the depth of each point of the scene with a size between 10 cm^2^ and 1 m^2^. The results of the reconstruction are good, but such a device is too prohibitively costly and complicated to be a viable solution for field use.

Thus, we focused our research on a common approach in computer vision : stereovision, also called Shape from Stereo. This 3D imaging technique was introduced by [1] in the early 1980s and detailed in [2]. It consists in the acquisition of a pair of images of the same scene by two cameras from different angles. These two cameras are spaced by a distance called “base”. Then, based on the pinhole camera model and epipolar geometry [3], the depth is determined from the disparity (difference between the position of an object viewed from multiple angles). This measure of disparity is the main difficulty for smooth functioning of this technique and depends on the choice of the base between cameras and their tilt angles. Indeed, the larger the base is, the more accurate the measure will be, but there will be more occlusions (a point on the scene viewed by a camera is not necessarily viewed by the other). These occlusion problems do not allow us to obtain good results due to the kind of scene where this phenomenon often happens (crops). A 3D reconstruction technique that frees itself from occlusion problems is necessary. We can group 3D reconstruction techniques into three large families : geometric approaches, photometric approaches and those based on the physical properties of the acquisition system. Geometrical approaches are based on the knowledge of the scene structure and the internal and external parameters of the cameras used. Stereovision technique is part of this approach. In the case of photometric approaches, the principle is the evaluation of a pixel's intensity to obtain 3D information as in the case of the method known as Shape from Shading [4]. Finally, many techniques of the previous techniques are based on the pinhole model; the third approach uses a real optical system. The main difference is that instead of considering a perfect projection of all points of the scene onto the image plane, only some of these points are projected correctly. This phenomenon comes from a limited depth of field that will be explained later.

The Shape from Focus technique (SFF) [5] or Depth from Focus is based on this depth of field. This technique is used to solve our problem of 3D acquisition of a scene with strong occlusions. This is a passive and monocular technique that provides a depth map of a scene based on a stack of 2D images. This stack is obtained by varying the camera/object distance (*d_co_*) according to a defined step where, for each step, an image is acquired in order to scan the entire scene. A focus measure is calculated for each pixel of each image according to a local window, and the spatial position of the image where this measure is maximal is determined. This image position allows linking each pixel to a spatial position to obtain the depth map. The main drawbacks of this method are the need for a textured scene, because the focus measure is based on the high frequency content of the scene, and a large number of acquired images.

### 2.2. Optical Principle

To better understand the physical principles governing the creation of sharp or blurred image and the acquisition process of image stack, a brief reminder of the optical properties is proposed.

**Figure 1.**
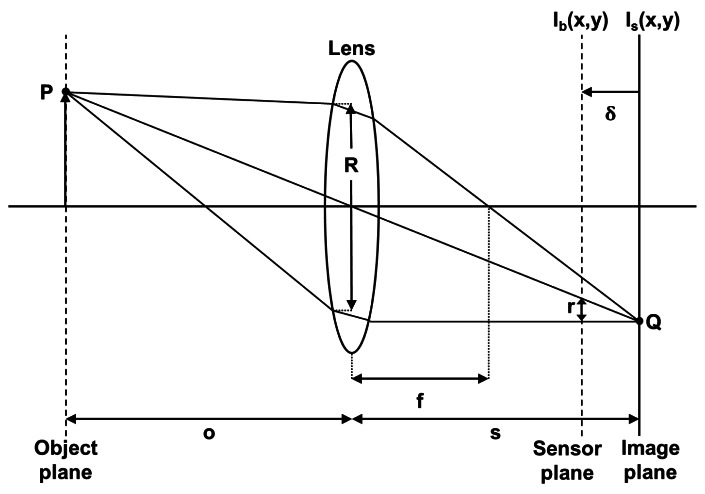
Sharp and unsharp image formation.

In Figure 1, all the rays emitted by the point P of an object and intercepted by the lens are refracted by this one and converge at point Q in the image plane. The equation for the focal lens depending on the *d_co_* distance and lens/image plane is:

(1) $$\frac{1}{f}=\frac{1}{o}+\frac{1}{s}$$

Each point of the object is projected onto the image plane at a single point and leads to the formation of the image *I_s_*(*x*, *y*). If the image plane does not merge with the sensor plane, where the distance between them is *δ*, the energy received from the object by the lens is distributed on the sensor plane in a circular shape. However, the shape of this energy distribution depends on the shape of the diaphragm aperture, considered circular. The radius of this shape can be calculated by:

(2) $$r=\frac{\delta .R}{s}$$

where *R* is the aperture of the lens.

The blurred image *I_b_*(*x*, *y*) formed on the sensor plane can be considered as the result of a convolution between a sharp image *I_s_*(*x*, *y*) and a blur function *h*(*x*, *y*).

(3) $$I_{b}\;(x,y)=I_{s}\;(x,y)*h\;(x,y)$$

This blur function can be approximated by a low pass filter (Equation (4)).

(4) $$h\;(x,y)=\frac{1}{2\pi \sigma_{h}^{2}}\exp^{-\frac{x^{2}+y^{2}}{2\sigma_{h}^{2}}}$$

The spread parameter *σ_h_* is proportional to the radius *r*, thus the larger the distance *δ* between the image plane and the sensor plane is, the more high frequencies are cut. In consequence, we obtain a blurred image.

**Figure 2.**
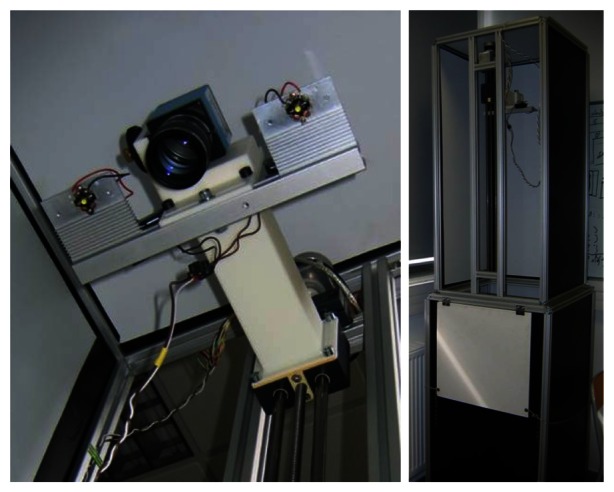
Acquisition system.

However, by using a real optical system, the object plane is not a plane but an area where the projected image will be sharp. This area corresponds to a depth of field (Figure 2) and can be calculated by the following equations:

(5) $$\text{DoF}=\frac{2A.C.F^{2}.D.\left(D-F\right)}{F^{4}-A^{2}.C^{2}.{\left(D-F\right)}^{2}}$$

The depth of field depends on four parameters: *d_co_* distance (*D*), aperture (*A*), focal length (*F*) and radius of the circle of confusion (*C*). The choice of all these parameters will affect not only the depth of field (DoF) but also the field of view (FoV) available.

(6) $$\frac{w}{W}=\frac{h}{H}=\frac{F}{D}$$

where *w* and *h* are the width and height of the sensors, *W* and *H* are the width and height of the scene considered and correspond to the available field of view following the optical configuration.

(7) $$W=\frac{w.D}{F}$$

(8) $$H=\frac{h.D}{F}$$

The focal length and the aperture value (F-Number) will therefore depend on the kind of lens used. Also, the diameter of the circle of confusion and the dimensions of sensor will depend on the kind of camera used. For the diameter of the circle of confusion, we will consider the value of the width of a pixel. Table 1 gives an example of values of field of view and depth of field obtained for different lenses associated with a ½ inch camera sensor and a pixel width of 4.65 μm.

In conclusion, the depth of field decreases when the focal length or the aperture value increases. In the same way, it increases when the diameter of the circle of confusion or the *d_co_* distance increases. This depth of field is directly correlated with the depth resolution of the 3D reconstruction.

## 3. Acquisition System

The 2D image stack is performed by a displacement of the depth of field in order to scan the considered scene. According to Figure 1, the displacement can be obtained in several ways:

– Displacement of optical system
– Displacement of object
– Displacement of lens (zoom)

The last kind of displacement has the drawback of changing the depth of field, which must be constant between each acquisition, and leads to a non-constant magnification. These magnification effects are explained in details in [6].

Therefore, there remains the possibility of moving the optical system or the object but the latter solution is not possible for crops. We selected the displacement of the acquisition system to vary the focal plane. As explained in [7], by varying the *d_co_* distance following a constant step and keeping the optical parameters fixed (aperture and focal length), a constant magnification appears during the acquisition.

In order to perform displacement of the optical unit, we use the system of Figure 2. The optical unit is centered on the desired field of view and two power LEDs are used to illuminate the scene. A stepper motor is coupled to a linear displacement with trapezoid screw and allows moving the optical unit incrementally and precisely. The motor control is carried out by a micro-controller associated with a power card. Both this card and the LEDs are powered by a 12 V battery with a 12 V/5 V supply for the micro-controller card. The acquisition system is transportable and self-powered, which allows acquisitions in the field. The camera and the micro-controller are both connected to a rugged computer via USB and controlled by an interface coded in C++.

The choice of step corresponding to the displacement between each acquisition depends on the depth of field. A size of step corresponding to the depth of field involves a better accuracy of the focus measure because the sharp areas are not the same between two successive images.

The optical unit includes a CCD camera with a ½ inch sensor with a resolution of 1280 by 960 pixels and the size of these is 4.65 μm. We use a 50 mm lens with 1.4 aperture, which allows a value depth of field of 5 mm for a distance of one meter.

**Figure 3.**
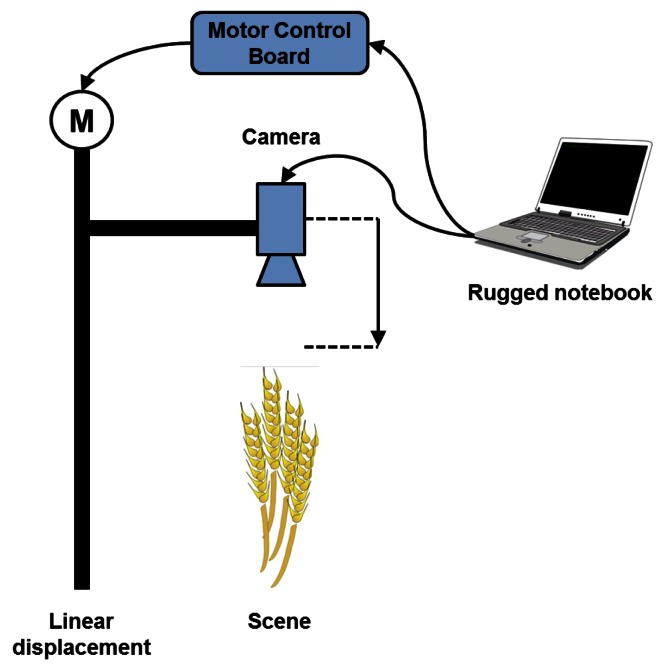
Acquisition process.

A schematic overview of our acquisition process is presented in Figure 3.

## 4. Image Processing

### 4.1. Calibration

Once the images have been acquired by the system, several processing operations are performed to make them usable.

No camera is perfect, so it must be calibrated in order to correct various distortions induced by the lens used. To do this, we used the toolbox “Camera Calibration Toolbox for MATLAB” [8]. Based on an image stack of a calibration pattern acquired from different viewpoints, the transformation matrix is obtained to correct distortions. Of course, distortions vary according to the lens quality and the focal length. Thus, the longer the focal length is, the less distortion there is. With the use of a 50 mm lens for our system, these distortions are almost quite null.

As explained previously, a problem with this acquisition process is the magnification effect linked to the displacement. This magnification induces several undesirable effects like the decrement of the field of view, which causes a difference between the images of the stack that must be similar for image processing.

Normally, this magnification is not considered because the displacement is very small between each acquisition (a few microns) as in microscopy. However, the macroscopic aspect of our application requires a correction of the magnification. Several solutions to correct or overcome this phenomenon can be found in the literature. For example, [6] proposes to compensate the magnification by changing the focal length in each step in order to always view the same areas. The major drawback of this technique is a non-constant depth of field because this one depends on the focal length. [9] recommends the use of a telecentric lens to ensure a constant magnification irrespective of the *d_co_* distance. This kind of lens is not suitable for our scene because it is designed for the visualization of small objects.

**Figure 4.**
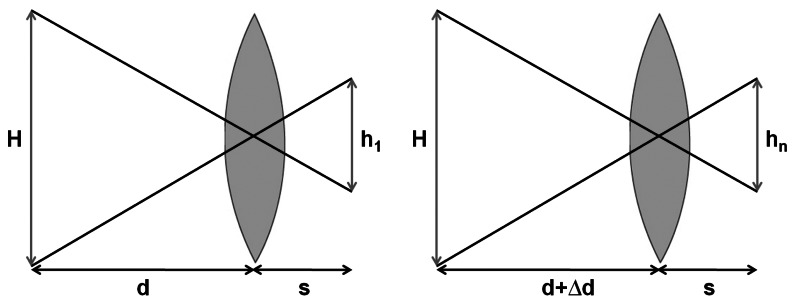
Optical scheme for the 1st and the nth image.

For our application, the step of displacement of the optical system is the same between each acquisition. Schematically, we are in the case shown in Figure 4 where *d* is the *d_co_* distance known because the system is calibrated and Δ*d* is the value of the step between two acquisitions.

Thus, we can determine the magnification ratio *^hn^*/*_h_*_1_by using the intercept theorem.

(9) $$\frac{h_{n}}{h_{1}}=\frac{d}{d+\Delta d}$$

This ratio is used to crop the images except for the last image of the sequence. Indeed, this one represents a totally visible scene in all the other images. When all the images are cropped, scaling is applied to recover a single size for all images according to the size of the last one. Afterwards, we obtain an image stack with the same size and representing an identical scene. In practice, there is a last detail to correct, which is the small displacement of the optical center between each acquisition due to the vibrations involved by the movement of the system. An image registration must be applied to the images to match their optical center. For this, we use the phase correlation method detailed in [10] and used in context of depth from defocus reconstruction by [11]. This technique is based on the Fourier shift property: a shift between two images in the spatial domain results in a linear phase difference in the frequency domain.

This allows to estimate the relative translation between two images and is composed of several steps.

1. Application of a Hamming window over the images to avoid the noise involve by the edge effects.
2. Calculation of Discrete Fourier Transform of the two previous modified images.
3. Determination of cross-power spectrum *R*, where *F* is the Fourier transform of the first image and *G* is the Fourier transform of the second image.
  (10) $$R\left(u,\upsilon \right)=\frac{F\left(u,\upsilon \right).\bar{G}\left(u,\upsilon \right)}{\left|F\left(u,\upsilon \right).\bar{G}\left(u,\upsilon \right)\right|}=e^{-j2\pi \left(ut_{x}+\upsilon t_{y}\right)}$$
4. Application of inverse Fourier Transform to the matrix *R*. The result is an impulse function that is approximately zero everywhere except at the displacement. The coordinates *t_x_* and *t_y_* of this impulse is used to register the images.
5. Repetition of all these steps for all images of the sequence.

Several image registration methods can be used for our problem because this one is a non-complex case. Indeed, only a translation could appear between two successive images. We use the phase correlation method because it is fast and easy to implement. It is particularly useful for image registration of images taken under varying conditions of illumination. These variations of illumination could appear with shape from focus when we take image without control of the acquisition environment. When the image registration is completed, the image stack can be used to apply the focus measure operators.

### 4.2. Focus Measure

As explained before, we can consider the blur image formation by the convolution of a sharp image and a blur function. This blur function can be approximated by a low-pass filter, thus the sharper the image is, the more high frequencies are contained in the image. These frequencies correspond to very contrasting textured areas. To measure the sharpness means to quantify these high frequencies.

We can consider this measure like the following function:

(11) $$f_{i}\left(x,y\right)=max_{i}\left(FM_{i}\left(x,y\right)\right)$$

where *i* = 1 …*N*, *N* is the number of images of the sequence, *FM_i_*(*x*, *y*) is the focus measure applied in a local window around pixel (*x*, *y*) for the *i^th^* image.

This local approach consists in the measurement of the sharpness of each pixel by applying a local operator to all images of the sequence. The size of the measurement window is an important choice with a strong impact on the precision of the depth map. Indeed, the smaller the window size is, the more prone to noise is the measure. On the other hand, the larger the window size is, the smoother the depth map will be, and will involve problems for the discontinuous areas.

We find many kinds of measures in the literature. First, there are the differential measures. As explained in the section on optical phenomena, the more an image becomes blurred, the wider is the diameter of the blurred shape supposed to represent a point of the scene. This blurred shape leads to the distribution of energy of a pixel on all adjacent pixels. There are many differential measures, for example, the Brenner gradient [12], the energy of gradient or Laplacian [13]. Among the most used operators in Shape from Focus, there are the Tenenbaum gradient (Tenengrad) [14] and the sum of modified Laplacian (SML) [5]. There also the measures of contrast [15]. Indeed, the more blurred an image is, the bigger the energy distributed on a neighborhood is. Thus we can quantify the local contrast that is bigger when an image is sharp. The computation of the local variance around the pixel allows to measure the variation of the gray levels of this neighborhood [16]. High variance is associated with a sharp local neighborhood, while a low variance means that the neighborhood is not sharp. Another kind of measure is based on the histogram because a sharp image contains more gray levels than an image that is not sharp. Thus, work [17] suggests to use the difference between the maximum gray level and the minimum as a measure of focus. As explained previously, a sharp area contains more high frequencies than a blurred area, so a lot of measures are based on the frequency domain. This is the case of [18] with the Fourier transform, [19] with discrete cosine transform and wavelet transform [20]. Finally, we find the 3D focus measures that consist in the use of the neighborhood in the current image but also the same neighborhood in the previous and next images of the sequence. This kind of measure is based on a principal component analysis associated with a spectral transformation as in [21].

In our application, we use the Tenengrad variance measure [22]. Since a sharp and textured image has more pronounced edges, it seems natural to use an edge detector to calculate the sharpness. Moreover, we can find a comparative study of different operators in [23] and the Tenengrad is considered as the best operator. The amplitude of the gradient is calculated by Equation (15), where *G*(*x*, *y*) is the convolution between image *I*(*x*,*y*) and Sobel operators *S_x_* and *S_y_*.

(12) $$S_{x}=\left(\begin{array}{lll} -1 & 0 & 1\\ -2 & 0 & 2\\ -1 & 0 & 1 \end{array}\right)\;S_{y}=\left(\begin{array}{lll} 1 & 2 & 1\\ 0 & 0 & 0\\ -1 & -2 & -1 \end{array}\right)$$

(13) $$S\left(x,y\right)=\sqrt{{\left[G_{x}\left(x,y\right)\right]}^{2}+{\left[G_{y}\left(x,y\right)\right]}^{2}}$$

The variance of Tenengrad is given by:

(14) $$FM_{\text{tenvar}}\left(i,j\right)=\sum_{x=i-N}^{i+N}\sum_{y=j-N}^{j+N}{\left[S\left(x,y\right)-\bar{S}\right]}^{2}$$

where $\bar{S}=\frac{1}{N^{2}}\sum_{x=i-N}^{i+N}\sum S\left(x,y\right)$and *N* is the size of the neighborhood.

We obtain a curve for each pixel where the position of the maximum represents the image for which this pixel is sharp.

Finally, we use an approximation method to refine the result of the position of the sharpest pixel.

**Figure 5.**
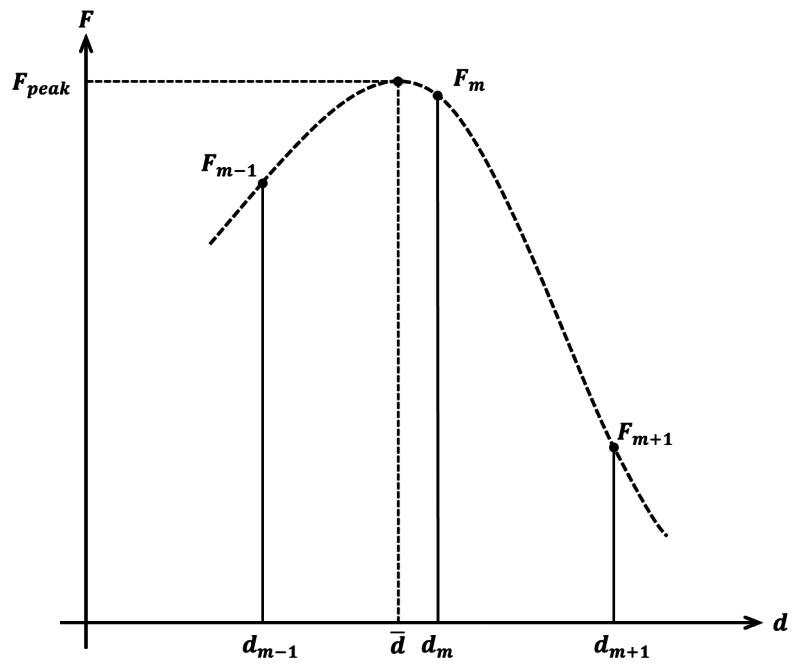
Gaussian approximation.

We use a Gaussian interpolation based on just three points of the focus curve (Figure 5) for a faster computation.

We look for *d̄* where the focus value is maximum (*F_peak_*). Three measures are used : F*_m_*_−1_, *F_m_* and *F_m_*_+1_.

(15) d¯=d+mlog(Fm−1)−log(Fm+1)2log(Fm−1)−4log(Fm)+2log(Fm+1)

*d̄* is the approximated position of the sharpest pixel.

If three points are not sufficient for a good approximation, we use a complete Gaussian curve fitting. It is slower than a three-point interpolation but more accurate.

### 4.3. Depth Map

**Figure 6.**
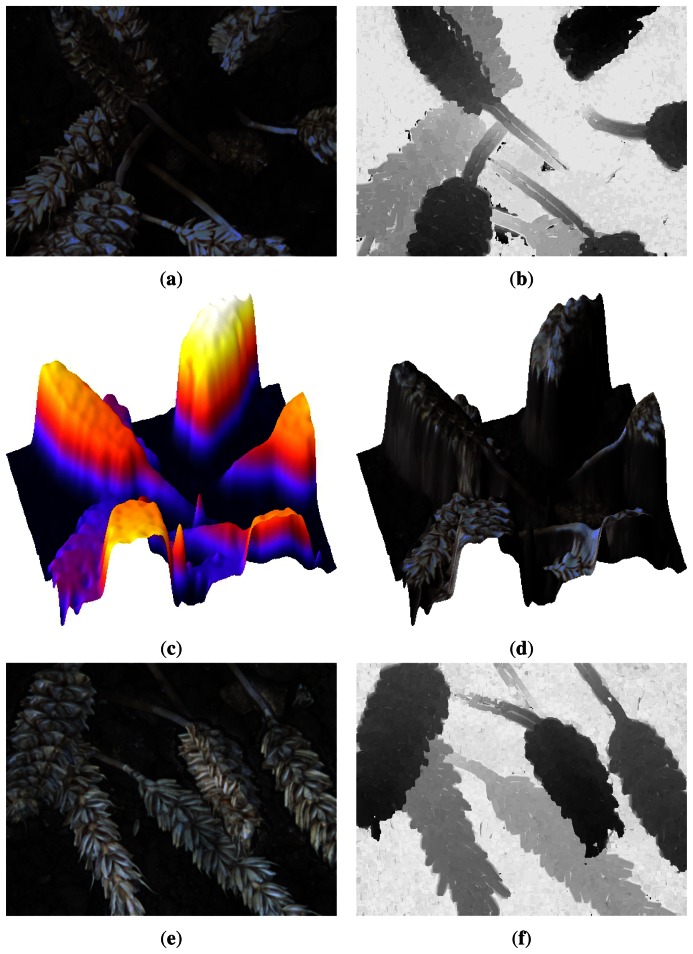

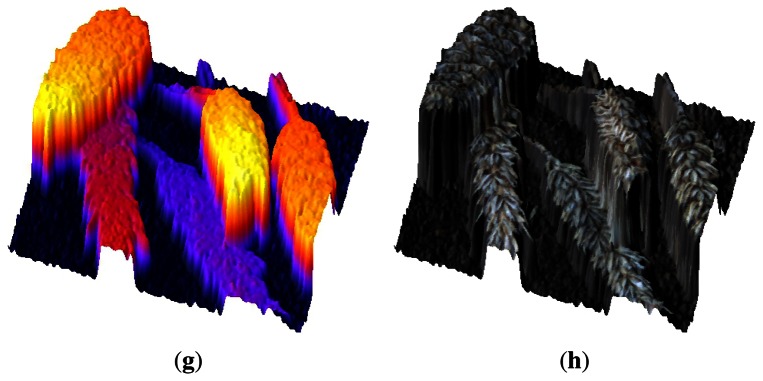
Merged images of two different sequences (**a**,**e**), associated depth maps (**b**,**f**) and 3D visualizations with (**d**,**h**) and without (**c**,**g**) texture mapping

When a focus value is assigned to each pixel of all the images, these results are used to obtain the depth information. Indeed, a curve is obtained for each point of the scene. Then, we estimate the maximum of this curve to determine in which image the point is the sharpest. Once this is performed for all the points, we associate a gray level to each point according to the sharp position to obtain the depth map (Figure 6). The primary purpose of these depth maps is the association with our previous research based on the automatic counting of wheat ears [24]. Indeed, the depth map allows us to distinguish the objects that are not located on the same spatial plane but overlapped in 2D images. The knowledge of spatial location of each pixel can also eliminate unnecessary information such as the floor of the scene. Thus, we realize a spatial segmentation to improve accuracy and rapidity of our post-processing. Moreover, with the focus measure values, we can create a merged image to retrieve a 2D image from the sequence. The depth map can be used to have a 3D visualization of the scene and the merged image is used to map texture on this 3D visualization. Two examples of these different results can be found in Figure 6.

## 5. Conclusions

This paper demonstrates the feasibility of using Shape from Focus technique at a macroscopic level. This 3D reconstruction method is suitable for the reconstruction of scenes with a random objects repartition. The registration problem is solved by the phase correlation method and a depth map can be created through the application of a focus measure. In our case, a focus measure based on the variance of Tenengrad provides good results because of the textured areas in our kind of scene.

The designed acquisition system is transportable and easy to use in the field. Moreover, this system is not designed to be mounted on an agricultural machine but in an autonomous vehicle dedicated to parameter acquisition in field. Future works will focus on improving the existing system and mainly on the optical part to improve the accuracy by decreasing the depth of field. An important point for any use of this acquisition method is the tradeoff between accuracy and the available field of view, because both are linked and depend on the user's needs or requirements.

The achievement of the depth information allows going further in the extraction of features necessary to characterize crops. The primary goal is to obtain an accuracy enabling the characterization of the number of grain per wheat ear to evaluate yield at an early stage.

## Figures and Tables

**Table 1.** Field of view and depth of field according to the kind of lens (values in millimeter).

D	25 mm f1.4			35 mm f1.6			50 mm f2		

	Width	Height	DoF	Width	Height	DoF	Width	Height	DoF
800	204.8	153.6	12.91	146.2	109.7	7.43	102.4	768	4.46
900	230.4	172.8	16.4	164.5	123.4	9.45	115.2	864	5.69
1,000	256	192	20.31	182.8	137.1	11.72	128	96	7.06
1,100	281.6	211.2	24.63	201.1	150.8	14.23	140.8	105.6	8.59
1,200	307.2	230.4	29.37	219.4	164.5	16.98	153.6	115.2	10.26
